# Heart transplantation in hereditary ATTR amyloidosis

**DOI:** 10.1186/1750-1172-10-S1-I22

**Published:** 2015-11-02

**Authors:** M Slama

**Affiliations:** 1 Hôpital Antoine-Béclère, Clamart, France

## 

Systemic amyloidosis related to mutation of TTR gene can be complicated with Familial Amyloid Cardiomyopathy (TTR-FAC), a severe and life threatening form of heart failure with preserved ejection fraction, with a poor prognosis. To date there is no proven effective specific treatment against TTR-FAC, and the usual treatments of chronic heart failure are either ineffective or contra indicated, except for diuretics. Progression of the disease results in reduction of physical capacity, repeated hospitalizations, increase in diuretics dose, and ultimately in refractory heart failure.

Cardiac transplantation has been performed in TTR-FAC since 2003, and reported in 40 patients in the literature with acceptable results in highly selected patients. The results of combined heart and liver transplantation appear to be similar or even better than for isolated heart transplantation, due to a “tolerance” phenomenon, with less cardiac rejection and less graft coronary artery disease.

We performed heart transplantation for TTR-FAC in 10 pts (9 men; mean age 59 years range 49 to 70), NYHA class II to IV, isolated in 6 and combined with a liver transplantation in 4. Patients had received a pacemaker (n=3) or a defibrillator (n=3) during their preoperative course. Mutations of patients were TYR77 in 5, SER24 in 1, GLU62 in 1, MET30 in 1. In hospital mortality was 40%.

Patients can be considered as good candidates to heart transplantation for TTR-FAC if they have symptomatic heart failure persisting under optimal medical therapy, disregarding their ejection fraction, normal pulmonary resistance, no major comorbidities, an acceptable physical and psychological condition, and limited neurologic involvement (ability to engage in a post-operative rehabilitation program is mandatory).

In the future, indications of combined heart and liver transplantations will have to be weighed against isolated heart transplantation in association with specific medical treatments (Tafamidis, or SiRNA).

